# Effector CD8 T Cell-Dependent Zika Virus Control in the CNS: A Matter of Time and Numbers

**DOI:** 10.3389/fimmu.2020.01977

**Published:** 2020-08-18

**Authors:** Loulieta Nazerai, Amalie Skak Schøller, Maria Rosaria Bassi, Søren Buus, Anette Stryhn, Jan Pravsgaard Christensen, Allan Randrup Thomsen

**Affiliations:** Department of Immunology and Microbiology, University of Copenhagen, Copenhagen, Denmark

**Keywords:** Zika virus, mouse model, adaptive immunity, T cells, B cells, memory, protection

## Abstract

Zika virus (ZIKV), a mosquito-borne flavivirus, came into the spotlight in 2016 when it was found to be associated with an increased rate of microcephalic newborns in Brazil. The virus has further been recognized to cause neurologic complications in children and adults in the form of myelitis, encephalitis, acute disseminated encephalomyelitis (ADEM) and Guillain Barre Syndrome in a fraction of infected individuals. With the ultimate goal of identifying correlates of protection to guide the design of an effective vaccine, the study of the immune response to ZIKV infection has become the focus of research worldwide. Both innate and adaptive immune responses seem to be essential for controlling the infection. Induction of sufficient levels of neutralizing antibodies has been strongly correlated with protection against reinfection in various models, while the role of CD8 T cells as antiviral effectors in the CNS has been controversial. In an attempt to improve our understanding regarding the role of ZIKV-induced CD8 T cells in protective immunity inside the CNS, we have expanded on previous studies in intracranially infected mice. In a recent study, we have demonstrated that, peripheral ZIKV infection in adult C57BL/6 mice induces a robust CD8 T cell response that peaks within a week. In the present study, we used B cell deficient as well as wild-type mice to show that there is a race between CXCR3-dependent recruitment of the effector CD8 T cells and local ZIKV replication, and that CD8 T cells are capable of local viral control if they arrive in the brain early after viral invasion, in appropriate numbers and differentiation state. Our data highlight the benefits of considering this subset when designing vaccines against Zika virus.

## Introduction

The emergence of Zika virus (ZIKV) as a threat to public health in the beginning of the 21st century caught everybody by surprise. ZIKV, a group B arbovirus belonging to the flavivirus genus along with Dengue (DENV), Yellow Fever (YFV) and West Nile (WNV) virus, was first isolated almost 70 years ago from a sentinel monkey in the Zika forest in Uganda (1). At that point and for several decades, ZIKV circulation in Africa did not raise any health concerns due to the relatively mild and self-resolving symptoms upon infection (1, 2). Geographically limited outbreaks, in 2007 in Micronesia and 2013 in French Polynesia, preceded the large outburst in microcephalic newborns in 2015 in Brazil, which ultimately led the WHO to classify ZIKV as a public health emergency of international concern from February 2016 until November 2016 (3–5). Extensive research has revealed that ZIKV has a wide tissue tropism and can cross the placental barrier of pregnant women, thus interfering with proper neurological development in the fetus (6–8). ZIKV has also been found to cause neuroinflammatory disorders in a fraction of otherwise healthy adults and children (9, 10). Amongst the most worrying features of ZIKV is perhaps the fact that infectious virus may persist in the testis and the virus may therefore be transmitted sexually (11). That, in combination with the virus’ predominantly asymptomatic nature and the current traveling patterns, can contribute to unrestrained viral spread also in countries where ZIKV is not endemic (12). An efficient vaccine is therefore much needed, and while there are several promising vaccine candidates in early stages of clinical testing, there is still none available on the market (5, 13). Elucidating correlates of protection is critical for a rational vaccine design and that requires extensive understanding of the immune responses elicited upon infection. For that reason, a number of animal models have been developed to study the biology behind ZIKV infection and protection (5, 14, 15). In mice, ZIKV is incapable of blocking the type I IFN response and viral replication following peripheral infection is limited; hence, the initial approach to study the infection in this species was to disrupt the type I IFN signaling pathway by either genetic manipulation or administration of IFNAR blocking Abs prior to infection (16–18). However, the role of type I IFNs in shaping adaptive immune responses, and particularly in shaping CD8 and CD4 T cell responses (19, 20), highlights the importance of including immunocompetent models to characterize ZIKV-induced T cell responses.

Generally, both immunocompetent and immunocompromised animal models have consistently shown the key role of CD4 T cells and humoral immunity in protective immunity (5, 21). On the other hand, the data regarding the contribution of CD8 T cells in restricting ZIKV replication, seems to vary significantly with the model employed. Following peripheral infection of adult mice, the results of several studies point to an antiviral role for CD8 T cells (22–25). In contrast, studies on i.c. challenged WT mice have indicated a very limited protective impact of CD8 T cells. Interestingly, in IFNAR deficient mice, in which peripheral infection also leads to CNS infection, quite contrasting results have been obtained. Adoptive transfer of virus-primed CD8 T cells prior to infection seems to reduce viral replication in the CNS (22); however, whether this is secondary to a reduced viral spread from peripheral sites or reflects a direct antiviral effect inside the CNS is not clear. Indeed, direct depletion of CD8 T cells from IFNAR deficient mice indicates that the CD8 T cell response may induce ZIKV-associated paralysis and augment pathogenesis (26, 27). Given the neurotropism of ZIKV and the fact that a fraction of postnatal ZIKV infections in humans may result in various forms of neuropathology including fatal encephalitis (9, 10, 28), we found it pertinent to determine whether CD8 T cells could contribute significantly to antiviral protection in the CNS and if that were the case, what are the factors deciding the disease outcome.

Induction of neutralizing antibodies (nAbs) has been the main focus when designing vaccines against flaviviruses. Nevertheless, the vaccination strategy has now started to shift due to increasing evidence pointing to a protective role of multifunctional CD4 and CD8 T cells, especially in cases where nAbs levels are low (29). An important consideration for vaccines against flaviviruses is the fact that they tend to circulate in the same geographical regions, so co-infection with more than one flavivirus is quite common. People infected with ZIKV may have been or may, at some point in their life, become infected with DENV and vice-versa, which raises concerns for antibody-dependent enhancement (ADE) of the secondary infection. Nevertheless, the latest study in rhesus macaques seemingly exclude the ADE scenario (30). Still, the presence of cross-reactive antibodies in relation to ZIKV traverse through anatomical barriers, such as those protecting the placenta and the brain, have not been sufficiently addressed yet. Interestingly, mouse studies have shown that cross-reactive DENV CD8 T cells, but not DENV-reactive Abs, can mediate protection against a successive ZIKV infection (31). Thus, there is undeniably still more to learn about the biology of flavivirus-induced CD8 T cells.

In this study, we add to the current understanding of CD8 T cells effector functions in the context of ZIKV infection of the CNS. We use adult B cell deficient and immunocompetent mice to model protective immunity following primary asymptomatic ZIKV infection and define the mechanisms of CD8 T cell infiltration in the brain following lethal ZIKV challenge. By performing drug- and antibody-based depletion of circulating cells and using a series of gene knockout mice, we show that ZIKV-specific effector CD8 T cells have the potential to limit viral replication at the site of infection, and that numbers and differentiation state of the circulating CD8 T cells are of the essence.

## Materials and Methods

### Mice

Female C57BL/6 (B6) wild-type (WT) mice, B6 albino mice and CD8 T cell deficient mice (H-2Kb/Db KO) on a B6 background were obtained from Taconic farms. B cell deficient mice (μMT/μMT, B6.129S2-Igh-6tm1Cgn/J), and IL-1R1 deficient (B6.129S7-Il1r1tm1Imx/J), were obtained from the Jackson Laboratory (Bar Harbor, ME, United States). IFNγ/Perforin double deficient (IFNγ/Pfp-/-), Perforin deficient (Pfp-/-), CXCR3 deficient (CXCR3-/-) and CXCL10 deficient (CXCL10-/-) mice on a B6 background were produced as previously described (32) and maintained locally. All mice used in this study were 7 to 14 weeks old and were housed under specific pathogen free (SPF) conditions at the ALAAC accredited animal facility at the Panum Institute (Copenhagen, Denmark). Mice coming from outside sources were allowed to rest for at least 1 week before entering an experiment.

### Virus Preparation and Quantitation

Zika virus, strain MR766 (Uganda, 1947), was obtained from American Type Culture Collection (ATCC) (Manassas, Virginia) and was propagated in Vero cells (ATCC CCL-81) grown in DMEM containing 10% FBS, glutamine and antibiotics (penicillin and streptomycin). The titer of the virus stock was determined based on the number of plaque forming units (pfu) in semi-confluent monolayers of Vero cells. Specifically, 10-fold serial dilutions of the virus stock were prepared and incubated for 2 h on Vero cell monolayers that were seeded a day earlier in 24-well plates. After the 2 h incubation, cells were overlayed with medium containing 0.9% methylcellulose and were further incubated for 5 days (37°C, 5% CO_2_). After fixation with 4% formaldehyde, cells were stained with 0.1% crystal violet for plaque visualization.

For quantitation of virus in the organs of mice, the organs were first homogenized in PBS to yield 10% suspensions and viral titers were subsequently determined as described above. The detection limit of the assay was 250 pfu/g of organ. For the experiments involving YFV, virus stock of strain YF-17D was produced and quantified as previously described (33).

### Immunization and i.c. Challenge

Mice were immunized by intravenous (i.v.) injection of 1 × 10^3^ pfu ZIKV MR766 in 300 μl. During viral challenge the mice were deeply anesthetized and 1 × 10^3^ pfu ZIKV MR766 in 30 μl was inoculated intracranially (i.c.). Health status and weight were monitored daily after i.c. challenge, and mice were euthanized when severe signs of illness along with a weight loss exceeding 25% of the initial weight were recorded. Immunization with YFV was done and mice were monitored as previously described (33).

### *In vivo* T Cell Depletion

The InVivoMab anti-mouse CD8a (YTS 169.4) purchased by BioXcell was used for *in vivo* depletion of CD8 T cells. Mice to be depleted were injected intraperitoneally (i.p.) with 200 μg of the antibody 1 day prior to i.c. challenge and with 100 μg of antibody 1 and 4 days post challenge. The FTY720 drug purchased by Sigma-Aldrich was used for depletion of circulating T cells. FTY720 was dissolved in the drinking water of mice to a concentration of 2.5 μg/ml and administered to them 2 days prior to i.v. infection and throughout the duration of the experiment. The efficiency of the cell depletion was confirmed by flow cytometric analysis of blood and/or splenocytes.

### IVIS SpectrumCT Analysis

Inflammation levels in the brain of Albino B6 mice, following i.c challenge, were detected by using *in vivo* Imaging System (IVIS SpectrumCT, PerkinElmer) and a fluorescent *in vivo* imaging agent (ProSense 750 FAST, NEV11171, PerkinElmer). At the day of imaging, the ProSense 750 FAST was reconstituted in PBS and each mouse was intravenously injected with 300 μl containing 4 nmol of the probe. 5–6 h post administration of the fluorescent probe, mice were transferred to the IVIS SpectrumCT (PerkinElmer) and scanned for fluorescence. During the scan, mice were kept under isoflurane anesthesia. Data obtained by IVIS analysis were subsequently analyzed with the “living image” software (PerkinElmer). The measured fluorescence was expressed as average radiant efficiency (p/s/cm^2^/sr)/(μW/cm^2^). Fluorescence measured on the back of each mouse served as background fluorescence and was subtracted by the fluorescence measured on the brain area.

### Single-Cell Preparation

Brains were aseptically removed after intracardial perfusion with 20 mL PBS. Mice were deeply anesthetized during this process via i.p. injection of avertin (2,2,2 tribromoethanol in 2-methyl-2-butanol, 250 mg/kg). Following the perfusion, brains were transferred to RPMI 1640 medium [supplemented with 1% L-glutamine, 1% penicillin, 1% streptomycin, 1% 2-mercaptoethanol (2-ME) and 10% fetal calf serum (FBS)]. Single-cell suspensions were obtained by pressing the brains through a 70 μm nylon cell strainer, followed by centrifugation for 10 min (400 × *g*, 4°C). Lymphocytes were separated on a 37% Percoll gradient during 20 min centrifugation (2800 × *g*, 20°C). Following centrifugation, the supernatant was aspirated and the remaining pellet was vortexed and washed twice in RPMI 1640 medium and finally resuspended in PBS containing 1% BSA and 0.1% NaN_3_.

Spleens were aseptically removed and transferred to Hanks Balanced Salt Solution (HBSS). Single-cell suspensions were obtained by pressing the spleens through a 70 μm nylon cell strainer, followed by centrifugation and two washes in HBSS.

Blood was collected in 1000 U/ml heparin/HBSS and erythrocytes were lysed using Gey-solution (NH4Cl, KHCO3, Phenol red) for 5 min. Following centrifugation, cells were washed twice in HBSS and finally resuspended in PBS containing 1% BSA and 0.1% NaN_3_.

### Flow Cytometry Analyses

Approximately 1–5 × 10^6^ brain/spleen/blood cells were transferred to U-bottom 96-well microtiter plates and were incubated for 20 min (4°C in the dark) with PE-conjugated H-2D^*b*^ tetramers for ZIKV E_294–302_ (34) and subsequently stained for an additional 20 min (4°C in the dark) for relevant cell-surface markers. Next, the cells were centrifuged, washed, fixed in 1% PFA and finally resuspended in PBS and stored at 4°C until flow cytometric analysis. Cell samples were analyzed using FACS LSRFortessa cytometer (BD Biosciences), and the data was analyzed using FlowJo software version 10 (Tree Star).

### Antibodies

The following fluorochrome-conjugated Abs, purchased from Biolegend as anti-mouse antibodies, were used for flow cytometry surface staining: αCD8–APC or PE/Cy7 or BV510, αCD4–FITC or APC/Cy7 or PE/Cy7, αCD44–FITC, αCD45.2– PerCP-Cy5.5, αCD11b–APC or FITC or BV786, αCD11c– BV421, αLy6C–PE, αNK1.1–FITC. To prevent unspecific binding, un-labeled anti-CD16/32 antibody was included in all staining setups.

### t-Distributed Stochastic Neighbor Embedding (t-SNE) Analysis of Flow Cytometry Data

CD45.2 positive, single cells were manually gated using FlowJo (v10) software and the events were downsampled to 60.000 for each group of interest. The groups of interest were concatenated to one file, and the t-SNE plugin in FlowJo was used to calculate a combined t-SNE plot. Microglial cells were identified as CD45.2^*int*^/CD11b^*int*^, macrophages as CD45^*hi*^/CD11b^*hi*^, NK cells as CD45.2^+^ and NK1.1^+^/CD4^–^, CD4 T cells as CD45.2^+^/CD4^+^ and CD8 T cells as CD45.2^+^/CD8^+^ and each population was visualized as a heat map on the tSNE plot. The microglial and macrophages population were further accessed for CD11c, Ly6C, and CD11b expression and displayed as overlaying histograms.

### Statistical Evaluation

GraphPad Prism Software (version 7) was used for the statistical analysis. For comparison of multiple data sets, a Kruskal–Wallis test (one-way ANOVA test, non-parametric) was initially performed and if groups differed significantly, pairwise comparisons were subsequently carried out using a non-parametric Mann-Whitney *U*-test. A *p*-value of <0.05 was considered evidence of a statistically significant difference.

## Results

### Similar Composition of Cellular Infiltrates in the Brain of WT and IL-1R1 KO Mice Following i.c. Challenge

**FIGURE 1:**
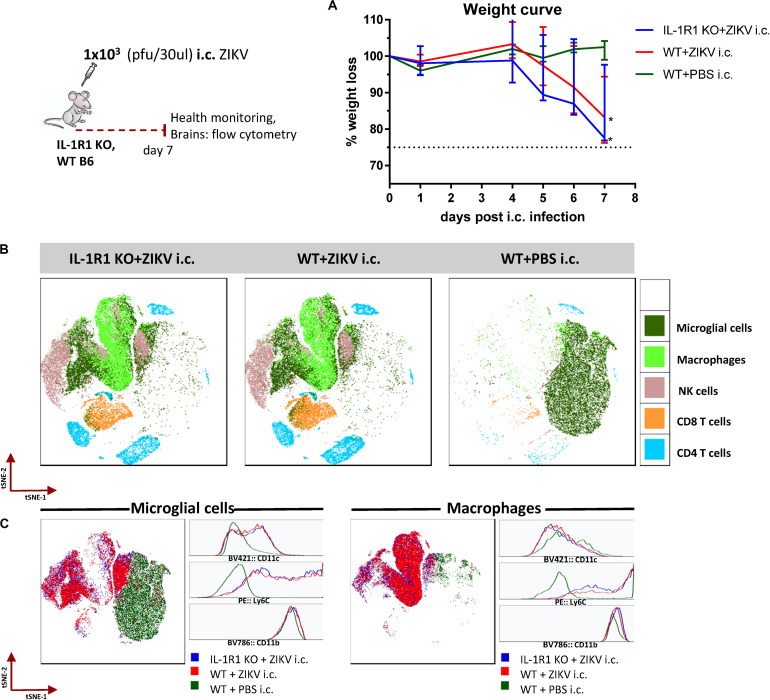
Composition of cellular infiltrates in the brains of IL-1R1 KO and WT mice following i.c. challenge with ZIKV. IL-1R1 KO and WT C57BL/6 mice were challenged with 1 × 10^3^ pfu ZIKV i.c. A group of naive mice injected i.c. with PBS was included for control. **(A)** Mice were weighed and monitored daily. **(B)** On day 7 post i.c. challenge, brains were removed and processed for flow cytometry. The flow cytometry data were analyzed and displayed as t-SNE plots to enable the identification of microglial cells (CD45.2^*int*^/CD11b^*int*^), macrophages (CD45^*hi*^/CD11b^*hi*^), NK cells (CD45.2^+^/NK1.1^+^/CD4^–^ ), CD8 (CD45.2^+^/CD8^+^/CD4^–^ ) and CD4 T cells (CD45.2^+^/CD4^+/^CD8^–^ ) based on the expression of relevant surface markers. **(C)** The population of microglial cells and macrophages are displayed as t-SNE plots along with histograms indicating the expression levels of CD11c, Ly6C and CD11b for each group. The weight curve depicts the group medians and ranges. *n* = 4–5/time point. The stippled line on the weight curve indicates the humane endpoint of 25% weight loss (compared to initial weight). The results are representative of two independent experiments.

In a previous report, we have shown that direct administration of ZIKV into the brain of adult wild-type (WT) C57BL/6 (B6) mice induces a lethal encephalitis (35). We were therefore interested in elucidating the composition of the cellular infiltrates in the brains of WT mice following intracranial (i.c.) infection with ZIKV. In light of data suggesting a critical role of IL-1 receptor signaling in controlling infection with West Nile virus (WNV) (36–38), an emerging flavivirus like ZIKV, we additionally wanted to assess ZIKV disease manifestation and cellular infiltration in the brains of IL-1R1 KO mice. To that end, adult IL-1R1 KO and WT mice were infected i.c. with ZIKV and their health and weight were monitored for a week (Figure 1). WT mice, challenged i.c. with PBS were included as controls. The course of the infection in IL-1R1 KO and WT mice was similar, and from day 5 onward both groups experienced acute weight loss and clinical disease (Figure 1A). On day 7 post i.c. infection, brains were removed and processed for flow cytometric assessment of the expression of relevant markers for microglia and infiltrating mononuclear leukocytes. The flow cytometry data were analyzed and displayed as tSNE plots where clusters of cells expressing similar markers were visualized (Figure 1B). The cellular populations in the brains of both IL-1R1 KO and WT mice challenged i.c. with ZIKV were found to be similar and consisted of microglial cells, monocyte/macrophages, NK cells, CD4 and CD8 T cells, while from the brains of WT mice receiving just PBS we recovered predominantly microglial cells. When looking closer at the populations, we observed that the microglial cells (CD45.2^*int*^/CD11b^*int*^), and monocyte/macrophages (CD45^*hi*^/CD11b^*hi*^) of ZIKV infected IL-1R1 KO and WT mice were activated, expressing high levels of Ly6C and for microglia also CD11c, which was not the case for mice receiving PBS (Figure 1C). Overall, these results indicate that IL-1 signaling is not critical in murine ZIKV infection and that upon i.c. challenge the cellular infiltrates consist primarily of inflammatory monocytes and activated microglial cells, but also NK and T cells.

### Kinetic of T Cell Infiltration in the Brain of Adult WT Mice Following i.c. Challenge

**FIGURE 2:**
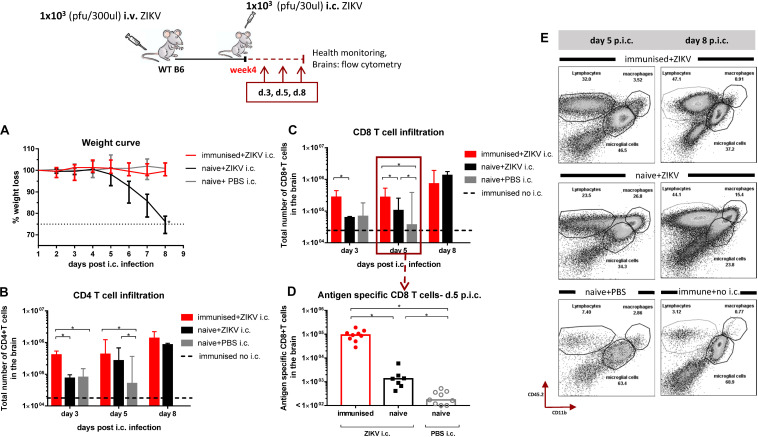
Kinetic of T cell infiltration in the brain following i.c. challenge with ZIKV. WT C57BL/6 mice were inoculated with 1 × 10^3^ pfu ZIKV i.v. and 4 weeks later, these mice along with naive controls, were challenged with 1 × 10^3^ pfu ZIKV i.c. A group of naive mice challenged i.c. with PBS and a group of immunized mice not challenged were also included for controls. Mice were weighed and monitored daily **(A)** and on days 3, 5, and 8 post i.c. challenge, brains were removed and the total number of CD4 T cells **(B)** and CD8 T cells **(C)** were determined via flow cytometry. For day 5 post i.c. the levels of ZIKV-specific CD8 T cells **(D)** were also determined. Representative flow plots of the cellular composition of infiltrating cells on days 5 and 8 post i.c. are included **(E)**. The results represent the group medians ± ranges and, for day 5 post i.c, are pooled from two independent experiments. The weight curve depicts the group medians and ranges. *n* = 4–5/time point. The stippled line on the weight curve indicates the humane endpoint of 25% weight loss (compared to initial weight). Each dot represents one animal, *p < 0.05.

Our group has previously established an *in vivo* model to study protective immunity in the context of ZIKV infection in WT immunocompetent mice (35). We have shown that primary asymptomatic ZIKV infection in mice results in solid protection against subsequent lethal i.c. challenge. The protection was strongly associated with the induction of nAbs, while the induced CD8 T cells were found to have a backup function in cases where the levels of nAbs were insufficient for efficient viral control (35). We now wanted to further study the role of CD8 T cells and wondered how the cellular infiltration in the brain of mice following i.c. challenge was associated with the protection observed in immune mice. Therefore, adult WT mice were rendered ZIKV-immune by peripheral inoculation with low doses of ZIKV and were challenged i.c. 4 weeks later. Brains were collected 3, 5, and 8 days post i.c. and were checked for T cell infiltration by flow cytometric analysis (Figure 2). Naive mice receiving ZIKV or PBS i.c. as well as immune mice not challenged i.c. were included for comparison. We observed that both CD8 and CD4 T cells were equally recruited to the brains of immune mice as a result of i.c. challenge (Figures 2B,C). The numbers of recruited T cells were higher in the immune mice on days 3 and 5 after i.c. challenge compared to naive mice similarly infected i.c. with ZIKV. Additionally, the majority of the recruited CD8 T cells in the immune group were ZIKV-specific as confirmed by tetramer staining for the ZIKV E_294–302_ epitope on day 5 post i.c. (Figure 2D). By day 8 post i.c, immune and naive mice challenged with ZIKV i.c. had similar numbers of infiltrating T cells in the brain – nevertheless, disease was severe and irreversible in the naive group as it is indicated by the weight loss curve (Figure 2A).

**FIGURE 3:**
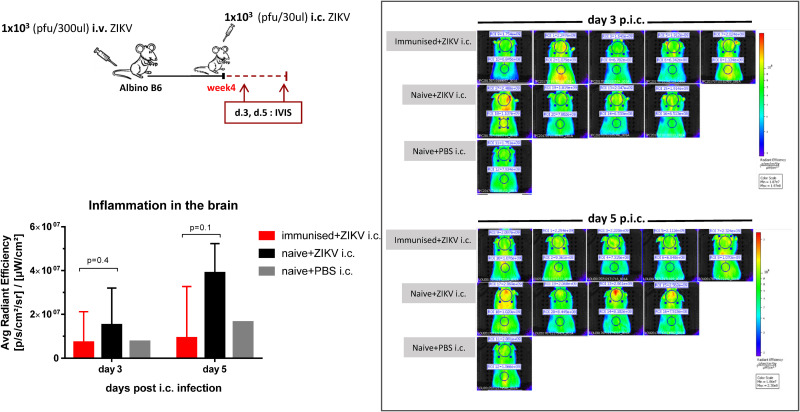
Inflammation levels in the brain. Albino C57BL/6 mice were inoculated with 1 × 10^3^ pfu ZIKV i.v. and 4 weeks later, these mice along with naive controls, were challenged with 1 × 10^3^ pfu ZIKV i.c. A group of naive mice injected i.c. with PBS and a group of immunized mice not challenged i.c. were also included for controls. Health status was monitored daily and on days 3 and 5 post i.c. challenge, mice were scanned for fluorescence in the IVIS SpectrumCT after administration of the fluorescent probe (ProSense 750, FAST) 6 h earlier. Representative images and scales indicating light intensity (ranging from dark blue for the least intense to red for the most intense) are included. The measured fluorescence on the back served as background fluorescence for each mouse and was subtracted from the fluorescence measured on the brain area. Fluorescence was expressed as average radiant efficiency. The results represent the group medians ± ranges. *n* = 1–5/time point. The experiment was also performed in BALB/c mice with similar results (data not shown).

In addition to direct killing of virus-infected cells, rapid influx of antigen-specific CD8 T cells can also contribute significantly to controlling the local inflammation that normally accompanies viral infections (39–41). We therefore set out to visualize and quantify the inflammatory activity in the brain of WT B6 albino mice on day 3 and 5 post i.c. using a cathepsin-activated fluorescent probe and *in vivo* Imaging System (IVIS) (Figure 3). As expected, the inflammation levels in the ZIKV-immune brains were found to be as low as the background inflammation levels of naive mice challenged with just PBS. In contrast, the inflammation levels in the brains of naive mice challenged i.c. with ZIKV tended to be high and to increase day after day post i.c. and probably until the point where the mice succumb to the infection. Consistent with this observation, analysis of the cellular infiltration on days 5 and 8 post i.c, when immune mice appear healthy while naive mice are clinically affected, revealed that although numbers of CD8 T cells were similar, monocytes/macrophage recruitment was markedly reduced in the ZIKV-immune mice (Figure 2E). Low inflammation levels in the brains of immune mice may be correlated with the early presence of ZIKV-specific CD8 T cells.

### Improved Viral Control in B Cell Deficient Mice Challenged i.c. During the Primary Effector Phase Correlates With the Presence of CD8 T Cells in CNS

**FIGURE 4:**
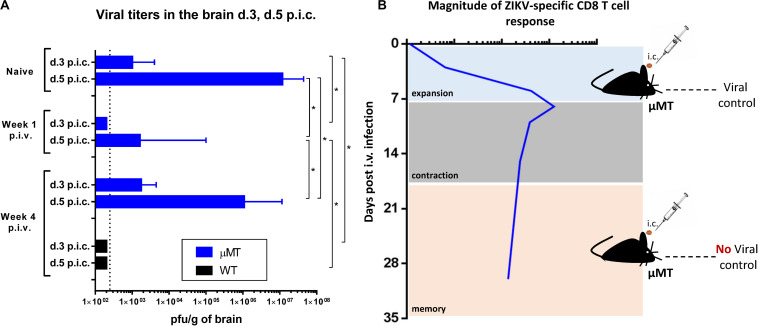
Improved viral control in B cell deficient mice when challenged i.c. during the effector phase. B cell KO (μMT) mice were inoculated with 1 × 10^3^ pfu ZIKV i.v. and 1 week or 4 weeks later, these mice along with naive μMT control mice, were challenged with 1 × 10^3^ pfu ZIKV i.c. WT C57BL/6 mice challenged with 1 × 10^3^ pfu ZIKV i.c. on week 4 post i.v. were also included for comparison. **(A)** Health status was monitored daily and on days 3 and 5 post i.c. challenge, brains were removed and viral titers were measured by a plaque assay. The detection limit for virus in brain was 250 pfu/g organ and is displayed as a stippled line. The results represent the group medians ± ranges. *n* = 4–5/group. *p < 0.05. **(B)** Graphical representation of the experimental setup in relation to the T cell levels at the time of i.c. challenge; T cell levels represent a schematic based on published results regarding numbers of virus specific CD8 T cells in the spleen of WT mice (35).

Neutralizing antibodies are excellent at controlling ZIKV infection on their own, so their presence in ZIKV-immune WT mice makes it challenging to study the potential contribution of CD8 T cells in protection. To monitor the role of CD8 T cells, we opted for using B cell deficient (μMT) mice which are unable to produce antibodies, but have an intact T cell response (42). From our previous studies (35), we know that the ZIKV-specific CD8 T cell response in WT mice peaks within a week post intravenous (i.v.) inoculation and that this population later contracts and reaches low but stable levels by week 4 post i.v. We hypothesized that the outcome of lethal challenge in μMT mice would largely depend on the size and effector capacity of the ZIKV-specific CD8 T cell population at the time of i.c. challenge. We therefore chose to peripherally inoculate the μMT mice with ZIKV and challenge them i.c. either 1 week or 4 weeks later. Naive μMT and immunized WT mice, both challenged i.c. with ZIKV, were included as controls. Brains were collected on days 3 and 5 post i.c. and viral loads were determined via plaque assay (Figure 4). We observed that μMT mice challenged i.c. during the effector phase (week 1) were better at limiting viral replication in the CNS compared to the μMT mice challenged i.c. during the memory phase (week 4), which reached significantly higher viral titers in their CNS. Still, the viral load in the latter group was significantly lower than in the naive control group, which implies that even in the memory phase, ZIKV-experienced T cells contribute to some extent to viral control. As expected, immunized WT mice were able to completely control the ZIKV infection.

**FIGURE 5:**
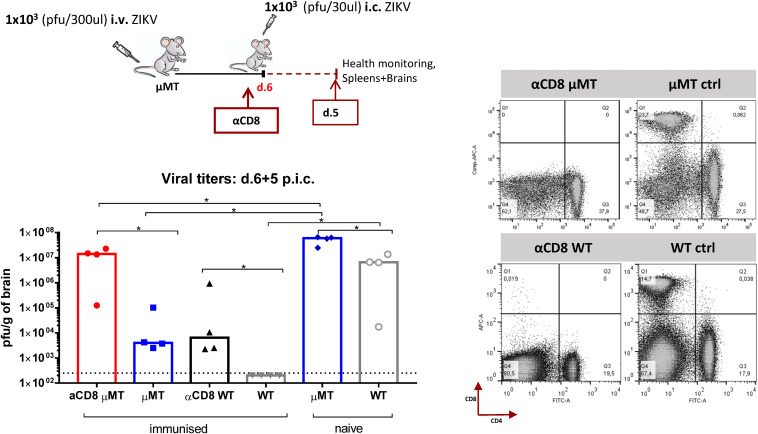
CD8 T cell depletion impairs viral control in the CNS of B cell deficient and WT mice. B cell KO (μMT) and WT C57BL/6 mice were inoculated with 1 × 10^3^ pfu ZIKV i.v. and 1 week later, these mice along with matched CD8 T cell depleted groups (αCD8 μMT, αCD8 WT), were challenged with 1 × 10^3^ pfu ZIKV i.c. Naιve μMT and WT mice were included for control. Health status was monitored daily and on day 5 post i.c. challenge, brains were removed and viral titers were measured by a plaque assay. The detection limit for virus in brain was 250 pfu/g organ and is displayed as a stippled line. Each dot represents an individual animal. The efficiency αCD8-depletion was assessed on splenocytes by flow cytometry; representative flow plots are included. The columns represent the group medians and are representative of two independent experiments, *p < 0.05.

Based on these findings, we had a strong indication that challenging the mice at the peak of the CD8 T cell expansion phase gave them their best chance to control lethal ZIKV infection. To further pinpoint that viral control is indeed interconnected with CD8 T cell effector function and numbers, we designed a similar experimental set-up as earlier, but this time μMT and WT groups depleted of CD8 T cells were also included, and viral titers in the CNS were assessed for day 5 post i.c. (Figure 5). We observed that the absence of CD8 T cells in both μMT and WT mice allowed ZIKV to replicate in the CNS at significantly higher levels compared to the corresponding undepleted μMT and WT mice. Still, virus loads in CD8 T cell-depleted WT mice did not reach the same high levels as in the brain of CD8 T cell-depleted μMT mice, which probably reflect the presence of nAbs in the former group (35).

**FIGURE 6:**
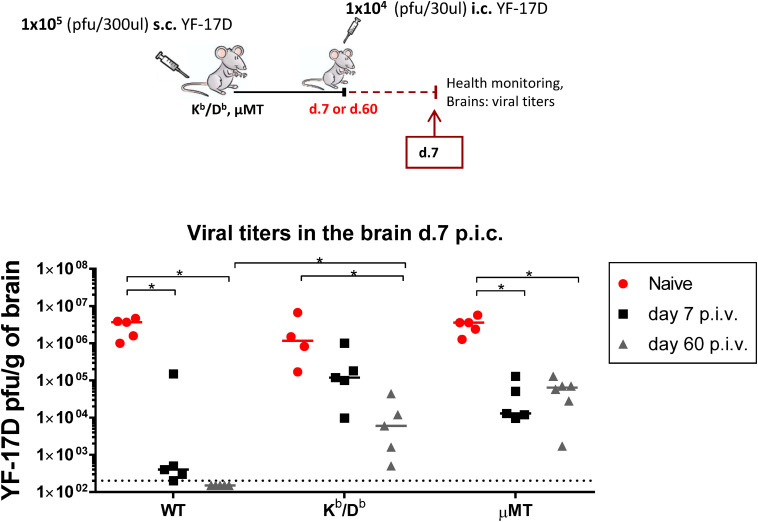
Viral control in CD8 T-cell deficient and B cell deficient mice when challenged i.c. with YF-17D virus during the effector and memory phase. WT C57BL/6, MHC class I CD8 T-cell deficient (K^*b*^/D^*b*^) and B cell deficient (μMT) mice were vaccinated with 1 × 10^5^ pfu YF-17D s.c. and 1 week or 8 weeks later, these mice along with naive control mice, were challenged with 1 × 10^4^ pfu YF-17D i.c. Health status was monitored daily and on day 7 post i.c. challenge, brains were removed and viral titers were measured by an immune focus assay. The detection limit for virus in brain was 200 pfu/g organ and is displayed as a stippled line. The results represent the group medians and each dot represents one animal, *p < 0.05.

To examine whether a similar pattern applied for another potentially neurotropic flavivirus, we infected MHC class I deficient mice lacking CD8 T cells and μMT mice lacking B cells with yellow fever virus (YF-17D, vaccine strain) peripherally and 7 (peak of the effector phase) or 60 (memory phase) days later, mice were challenged i.c. with a lethal dose of this virus (Figure 6). WT mice and matched naive mice for each KO group were included for control. Just like for ZIKV-infected mice, our results revealed that WT mice previously infected with YFV peripherally were quite resistant to homologous i.c. challenge already 7 days later, and that this early protection was insignificant in mice lacking CD8 T cells. Interestingly, CD8 T cells continued to play a substantial role in controlling YFV infection in the CNS even in the memory phase; the reason for this difference between the two viral infections could reflect differences in numbers and quality of available memory T cells as well as a difference in the rate of viral replication in the CNS.

### Accelerated Influx in the CNS as a Result of i.c. Challenge When Higher Numbers of Effector CD8 T Cells Are in Circulation

**FIGURE 7:**
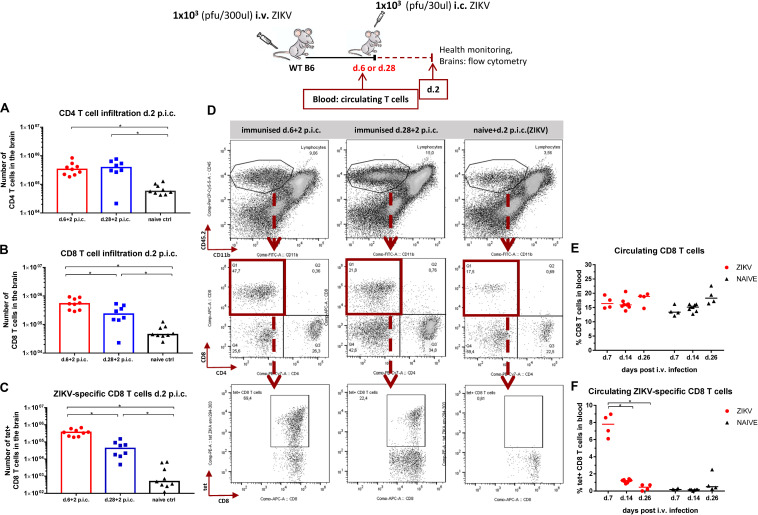
Accelerated influx in the CNS when higher numbers of effector CD8 T cells are circulating. WT C57BL/6 mice were inoculated with 1 × 10^3^ pfu ZIKV i.v. and either 1 or 4 weeks later, these mice along with naive controls, were challenged with 1 × 10^3^ pfu ZIKV i.c. Health status was monitored daily and on day 2 post i.c. challenge, brains were removed and the total number of CD4 T cells **(A)**, CD8 T cells **(B)**, and ZIKV-specific CD8 T cells **(C)** were determined by flow cytometry. Representative plots are included **(D)**. The levels of circulating CD8 T cells **(E)** and ZIKV-specific CD8 T cells **(F)** in the blood were assessed on days 7, 14, and 28 following peripheral i.v. inoculation. The columns represent the group medians and each dot represents one animal. Data are pooled from two independent experiments, *p < 0.05.

Having confirmed that the CD8 T cells during expansion phase are functionally equipped to contribute to viral control we wondered whether their recruitment to the brain following i.c. infection was more robust. We first assessed T cell infiltration patterns on day 5 post i.c, when virus levels in the brain were markedly different between the groups challenged i.c. during the expansion phase (week 1) and the contraction phase (week 4) (Figure 4), but we did not find any substantial differences in cellular recruitment (Supplementary Figure 1). We then hypothesized that, day 5 post i.c. was too late a time point to reveal critical differences in T cell influx, and we therefore decided to check cellular recruitment already on day 2 post i.c. Indeed, when challenged i.c. during the expansion phase of the virus-specific T cell response, mice experienced a higher influx of ZIKV-specific CD8 T cells in the CNS on day 2 post i.c. compared to the mice that were challenged i.c. during the contraction/memory phase (Figures 7B–D). The CD4 T cell recruitment to the brain, on the other hand, did not seem to differ regardless of the time point of i.c. challenge (Figure 7A). In Figures 7E,F, the levels of circulating CD8 T cells and ZIKV-specific CD8 T cells on days 7, 14 and 26 post i.v. are displayed and confirm the presence of a robust ZIKV-specific CD8 T cell population around week 1 post i.v. infection that has contracted significantly by week 4 post i.v.

### CD8 T Cell Deficiency During the Effector Phase Impairs Viral Control

**FIGURE 8:**
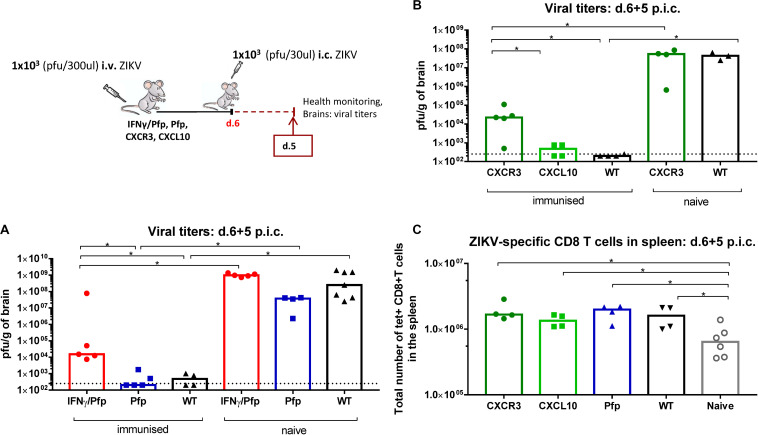
Impact of CD8 T cell deficiency in efficient viral control in the brain. **(A)** IFNγ/Perforin double-deficient (IFNγ/Pfp), IFNγ-deficient (IFNγ), and Perforin-deficient (Pfp) mice and **(B)** CXCR3-deficient and CXCL10-deficient mice, were inoculated with 1 × 10^3^ pfu ZIKV i.v. and 1 week later these mice along with naive controls, were challenged with 1 × 10^3^ pfu ZIKV i.c. WT C57BL/6 immunized mice were included as positive controls. Health status was monitored daily and on day 5 post i.c. challenge, brains were removed and viral titers were measured by a plaque assay. **(C)** The total number of ZIKV-specific CD8 T cells in the spleens of CXCR3-, CXCL10-, Pfp- deficient mice were assessed by flow cytometry. The detection limit for virus in brain was 250 pfu/g organ and is displayed as a stippled line. The results represent the group medians and each dot represents one animal, *p < 0.05.

We next wanted to further validate how the expanded circulating CD8 T cell population at the time of challenge affected protection levels by using genetically modified mice with targeted immune defects within the CD8 T cell compartment. We peripherally inoculated IFNγ/Perforin double-deficient (IFNγ/Pfp KO) and Perforin-deficient (Pfp KO) mice with ZIKV, and 1 week later we compared the outcome of lethal i.c. challenge on day 5 post i.c. to that in WT mice (Figure 8A). Naive mice were included as controls. We observed that the inability of CD8 T cells to produce both perforin and IFNγ led to impaired viral control with viral loads being significantly higher than in matched WT mice. The Pfp-KO group was not affected significantly and the protection was as good as in WT mice. That implies that secretion of IFNγ alone or in combination with perforin are required for CD8 T cells to control the infection.

We next wondered whether deficiencies in the CD8 T cell recruitment pathways would influence the viral loads in the CNS. We therefore immunized CXCR3-deficient and CXCL10-deficient mice with ZIKV 1 week before they were challenged i.c, and brains were removed and titrated on day 5 post i.c. (Figure 8B). We observed that optimal CD8 T cell recruitment to the site of infection is critical for protection since CXCR3-deficient mice could not achieve complete viral clearance in their CNS. Absence of CXCL10, which is one of the ligands for CXCR3, did not affect protection levels implying that other CXCR3 ligands might be more critical for the recruitment of CD8 T cells in the context of ZIKV infection. It should be noted that a significant systemic antiviral CD8 T cell response was observed in all gene deficient mice (Figure 8C).

**FIGURE 9:**
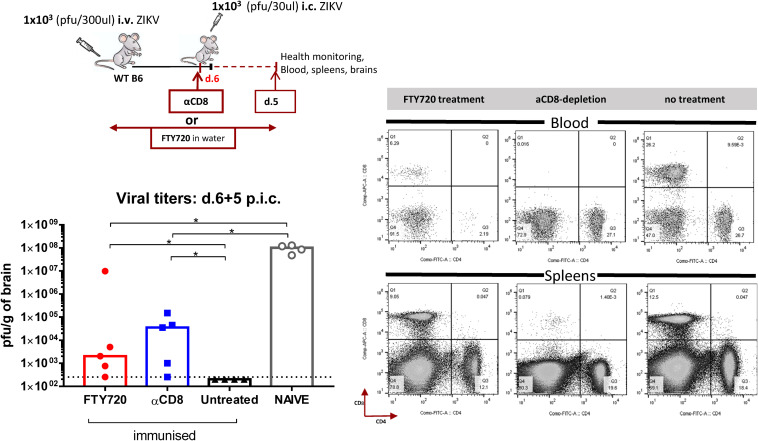
Effect of FTY720 treatment in protection during effector phase. WT C57BL/6 mice were inoculated with 1 × 10^3^ pfu ZIKV i.v. while FTY720 was administered in the water 2 days prior to i.v and for the whole duration of the experiment. Groups of immunized mice not receiving FTY720 (untreated), as well as a group receiving αCD8-depletion antibodies (αCD8), were included as controls. On day 6 post i.v, these mice along with naive controls, were challenged with 1 × 10^3^ pfu ZIKV i.c. Health status was monitored daily and on day 5 post i.c. challenge, brains were removed and viral titers were measured by a plaque assay. The efficiency of FTY720 administration and αCD8-depletion antibodies to reduce T cells was assessed on day 5 post i.c. in the blood and spleens by flow cytometry; representative plots are included. The detection limit for virus in brain was 250 pfu/g organ and is displayed as a stippled line. Each dot represents one animal. The columns represent the group medians, *p < 0.05.

To further assess the importance of recruited CD8 T cells in protection, we used the immunomodulatory drug FTY720 to reduce numbers of circulating T cells in the blood and check the effect this had on the course of i.c. challenge. FTY720 is a S1P1 antagonist, which blocks lymphocyte egress from lymphoid tissues thus leading to a decrease of peripheral lymphocytes (43, 44). Consequently, WT mice were immunized with ZIKV i.v. while FTY720 was administered in their drinking water starting 2 days prior to i.v. inoculation and until day 5 post i.c. when brains were removed and viral titers were measured (Figure 9). Groups of immunized and naive mice not receiving FTY720, as well as a group receiving αCD8-depleting antibodies, were included as controls. T cell status in the groups receiving FTY720 and αCD8-depletion antibodies was confirmed by flow cytometric analysis in blood and spleens. In agreement with literature, we found FTY720 treatment to reduce the circulating T cells in the blood, with no apparent impact on the T cells in the spleen (45). Depletion of CD8 T cells via antibodies removed CD8 T cells both from lymphoid tissues and from the circulation, while the CD4 T cell population remained unaffected. In accordance with our previous results, the viral loads in the brains revealed that, lack of circulating T cells in both the FTY720-treated and αCD8-depleted mice significantly impaired their ability for viral control.

### Peripheral CD8 T Cell Deficiency Affects CD8 T Cell Infiltration After i.c. During Effector Phase

**FIGURE 10:**
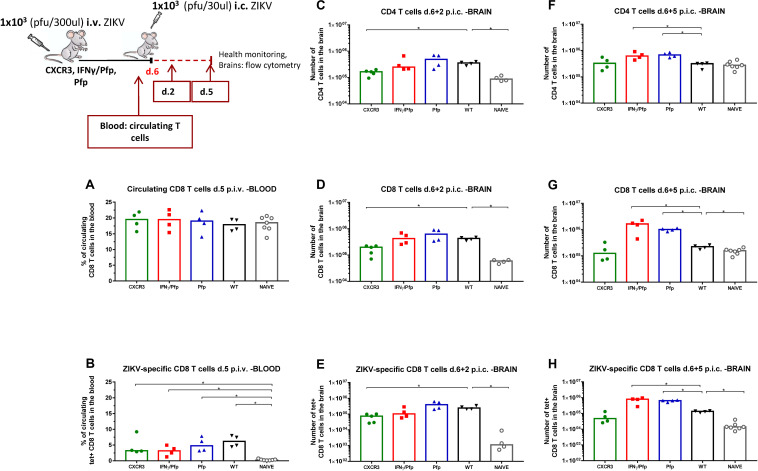
Impact of CD8 T cell deficiency on cellular recruitment to the brain. CXCR3-deficient, IFNγ/Perforin double-deficient, IFNγ-deficient and Perforin-deficient mice were inoculated with 1 × 10^3^ pfu ZIKV i.v. WT C57BL/6 immunized and naive mice were included as controls. On day 5 post i.v, the percentage of circulating CD8 T cells **(A)** and ZIKV-specific CD8 T cells **(B)**, were assessed in the blood by flow cytometry. On day 6 post i.v, mice were challenged with 1 × 10^3^ pfu ZIKV i.c. Health status was monitored daily and on days 2 and 5 post i.c. challenge, brains were removed and the total number of CD4 T cells **(C,F)**, CD8 T cells **(D,G)**, and ZIKV-specific CD8 T cells **(E,H)** were determined via flow cytometry. Each dot represents one animal. The columns represent the group medians, *p < 0.05.

Having correlated peripheral CD8 T cell deficiency with protection levels following i.c. challenge with ZIKV, we wanted to investigate whether the levels of recruited effector CD8 T cells in the brain are in agreement with that observation. Hence, CXCR3-deficient, IFNγ/Pfp double-deficient and Pfp-deficient mice were inoculated with ZIKV for 1 week and levels of recruited T cells were assessed on days 2 and 5 post i.c. and compared to WT mice (Figure 10). Naive mice were included for control. One day prior to lethal i.c. challenge the percentages of circulating CD8 T cells and ZIKV-specific CD8 T cells in the blood were assessed and found to be similar for all the groups (Figures 10A,B).

For day 2 post i.c, we observed that there was significantly reduced CD4 and CD8 T cell infiltration to the CNS of CXCR3-deficient mice compared to WT mice (Figures 10C,D). In addition, CXCR3-deficient mice had significantly fewer ZIKV-specific CD8 T cells recruited on day 2 post i.c. (Figure 10E). Interestingly, those differences became insignificant by day 5 post i.c. In combination with the viral titers data on the CNS of CXCR3-deficient mice (Figure 8B), it seems that failure of ZIKV-specific CD8 T cells to be rapidly recruited to the site of infection favors ZIKV replication, and, even if numbers of recruited ZIKV-specific CD8 T cells matched WT levels by day 5 post i.c, the course of the infection was already determined by the early impairment.

On day 5 post i.c, we detected increased ZIKV-specific CD8 T cell infiltration for IFNγ/Pfp KO and Pfp KO mice (Figure 10H). As observed earlier (Figure 8A), antiviral protection in the IFNγ/Pfp KO mice was reduced compared to WT mice and that can be attributed to the accumulation of CD8 T cells that are unable to mediate viral clearance. Therefore, numbers of recruited ZIKV-specific CD8 T cells are significantly elevated in this group at the late time-point, probably due to increased ZIKV replication in the CNS that in turn can fuel an overshoot in cellular recruitment. Regarding the Pfp KO mice, it is puzzling that we also see a small overshoot in T-cell infiltration on day 5 post i.c. even though viral control is not detectably impaired. Perhaps elimination of antigen expressing cells is slightly delayed despite good viral control.

## Discussion

The contribution of CD8 T cells in protection against ZIKV challenge is a controversial topic, particularly with regard to the brain (26). The induction of an adaptive CD8 T cell response is critical for the control and clearance of many viral infections and effector CD8 T cells can effectively handle secondary infections. Efficient CD8 T cell activation relies on appropriate cytokine signaling and in the case of viral infections is often dominated by type I IFNs (46). These signals ensure that dendritic cells are properly licensed and capable of presenting the viral antigen in secondary lymphoid organs, primarily the draining lymph nodes (47, 48). Therefore, animal models involving defects in type I IFN signaling present some limitations for understanding the arms of the immune system that are critical for protection.

In a previous study, we have established an *in vivo* model for ZIKV infection using adult immunocompetent mice with an intact IFN signaling pathway, and studied the immune response elicited by peripheral asymptomatic ZIKV infection and its contribution to protection against re-infection of the CNS (35). To allow viral replication in the CNS, we administered the virus intracranially, which in naive mice resulted in lethal infection characterized by acute weight loss and a high viral load in the CNS. Mice that were previously exposed to ZIKV via a peripheral route were found capable of successfully controlling viral replication in this organ, primarily via nAbs, CD4 T cells and, when nAbs levels were low, CD8 T cells (35). In contrast to this limited role of CD8 T cells in resistance to i.c. re-challenge, several studies have indicated the critical role of these cells on the course of peripheral ZIKV infection in mice with deficient IFNAR signaling (24, 49). This raised the question of whether CD8 T cells were simply relatively ineffective in controlling ZIKV infection of cells of the CNS (e.g., due to low MHC expression?) or the reason for the lack of a critical role of CD8 T cells in this organ was to be found in the spatiotemporal context i.e., perhaps the response was too little/too late (50). In the present report, we were therefore interested in exploring the mechanisms of CD8 T cell effector functions in the CNS and their contribution to ZIKV control.

We initially assessed the composition of the cells infiltrating the brain following a primary i.c. ZIKV infection in WT and IL-1R1 KO mice. In studies on a fellow neurotropic flavivirus, WNV, IL-1R signaling has been found critical for the control of viral replication in the CNS (36–38). We therefore set out to visualize the patterns of recruited populations to the CNS of WT and IL-1R1 KO mice. t-SNE analysis of extracted cells from both groups indicated that the composition of cellular infiltrates (microglial cells, macrophages, NK cells, T cells) was similar, suggesting that IL-1R does not play the same role for ZIKV control as it does for WNV. In the presence of ZIKV in the brain, microglial cells and macrophages displayed a distinct activated phenotype. Interestingly, these cell subsets did not exhibit the marked inflammatory profile in i.c. challenged ZIKV-immune or sham-infected mice, matching the limited fluorescence detected in the brain of these mice via IVIS analysis (following administration of a cathepsin-activated fluorescent probe). When we assessed the kinetic of the T cell recruitment to the brain of ZIKV-immune vs. naive WT mice, we observed an accelerated influx of both CD4 and CD8 T cells, which in the latter case included a large number of ZIKV-specific cells. When the circulating effector CD8 T cell population was reduced, either via FTY720 or via antibody-based depletion, antiviral protection was impaired. Therefore, unlike earlier studies all performed in IFNAR deficient mice (22–24), in which the increased viral load in the CNS could represent a consequence of augmented viral spread from peripheral sites, our results obtained in WT mice clearly demonstrate that the reduced viral replication in the CNS reflects *in situ* antiviral activity since there is no detectable peripheral ZIKV infection in WTs. This interpretation is further supported by the observations discussed in the following sections.

We and others have demonstrated that, upon peripheral asymptomatic infection, ZIKV elicits a robust antibody and CD8 T cell response that peaks within a week and then contracts (22, 35, 49, 51). The induced CD8 T cells are polyfunctional, as shown by their capacity to express IFNγ, TNFα and IL-2, and possess cytotoxic effector functions, as indicated by their degranulation capacity and release of granules containing granzyme B and perforin (22, 49, 51). Furthermore, ZIKV-specific effector CD8 T cells have been found to upregulate CD11a and CD49d, which, besides being markers of previous antigen encounter (52), together with CXCR3 are known to direct CD8 T cells to sites of infection (53, 54) including the CNS (55). To uncover the full antiviral potential of ZIKV-specific CD8 T cells in controlling a potentially lethal infection in the CNS, we first chose to work with B cell deficient mice that have an almost intact CD8 T cell compartment, but are unable to produce antibodies (42). We predicted that, under these conditions, the size and immediate effector capacity of the ZIKV-specific CD8 T cell population at the time of lethal challenge would determine the outcome of the infection. Indeed, when challenged i.c. around the peak of the ZIKV-specific CD8 T cell response (week 1), μMT mice displayed improved capacity to control viral replication in the brain. In contrast, when challenged i.c. during the memory phase (week 4), μMT mice displayed only delayed and limited capacity to control viral replication. The benefit of challenging mice around the peak of the ZIKV-specific CD8 T cell response was abrogated when CD8 T cells were depleted. As CD8 T-cell responses may be subject to increased contraction in B-cell deficient mice, it is important to note that efficient antiviral CD8 T-cell memory responses are intact in these mice (56). Furthermore, absence of CD8 T cells also resulted in increased ZIKV replication in the CNS of WT mice, when these were challenged early (week 1) after priming; still, the ability of WT mice to produce Abs secured these a better fate as would be expected from our earlier demonstration that virus-specific antibodies are present in the circulation already 6–7 days post infection (35). Overall, these data highlight that the CD8 T cells during expansion phase are functionally equipped to mediate viral clearance in the CNS. The capacity of CD8 T cells to control flaviviral infection in the CNS was further supported by observations in YFV-vaccinated mice, where effector CD8 T cells were found most essential for reducing viral replication when mice were challenged i.c. on day 7 post vaccination. Interestingly, during the memory phase CD8 T cells also contributed markedly to resistance to i.c. challenge with YFV, but not with ZIKV. Rather than reflecting a difference in the host response to these CNS infections, we attribute this to differences in the rate of viral replication following i.c. inoculation with the two virus strains we employed here (ZIKV MR766, YF-17D). Thus, maximal viral titers are reached already by day 5 post challenge in ZIKV-infected mice (35), while the maximal viral load in YFV-infected mice is not reached until day 7 after challenge (33), allowing for increased time to mobilize secondary effectors in the latter case.

Based on this, we decided to further test whether the superior capacity of effector ZIKV-specific CD8 T cells to mediate viral clearance early in the infection was due to their rapid recruitment to the brain. In point of fact, when mice were challenged i.c. during the expansion phase, ZIKV-specific CD8 T cells were found to robustly recruit to the site of infection already on day 2 post i.c. When the i.c. challenge was performed during the memory phase, ZIKV-specific CD8 T cells were also recruited to the brain on day 2 post i.c, but at a significantly lower rate, probably due to the limited numbers of circulating ZIKV-specific CD8 T cells at that time point. Additionally, memory CD8 T cells may require some time to acquire effector functions and enter the infected tissue (50, 57, 58). This fits nicely with the observation that by day 5 post i.c, both mice challenged at the expansion phase and in the memory phase, display similar numbers of infiltrating T cells. Nevertheless, it seems that the outcome of the infection is determined early after i.c. challenge, so insufficient or delayed recruitment of ZIKV-specific CD8 T cells to the site of infection might give a head start to the virus, which may then initially replicate uncontrollably.

Next, we were interested in understanding the mechanisms regulating effector CD8 T cell-dependent ZIKV control in the CNS. To that end, we employed a panel of genetically modified mice with targeted immune deficiencies within the CD8 T cell compartment and monitored the effect of lethal ZIKV challenge on protection. We found that, if the circulating CD8 T cells do not express the antiviral effector molecules IFNγ and perforin, even though they were recruited to the brain early and in sufficient numbers, they are not able to mediate viral clearance. Since a selective deficiency in perforin production did not significantly impair virus control in the CNS, we are led to assume that IFNγ alone or in combination with perforin are required for CD8 T cells to control ZIKV infection in this organ. Interestingly, numbers of recruited CD8 T cells in both IFNγ/perforin double deficient as well as perforin-deficient mice were found to be significantly higher than in WT controls 5 days after challenge, which could reflect that, in the absence of efficient virus control and/or just reduced killing of antigen-presenting cells (59) in the CNS there may be prolonged recruitment of antiviral effectors. Alternatively, this increased local recruitment of CD8 T cells in both double and perforin only knock-out mice could potentially have reflected a direct impact of these effector molecules in the regulation of the antiviral CD8 T-cell response (60). However, such an effect of the effector molecules has been questioned by others (61). Furthermore, as numbers of circulating CD8 T cells in these mice are not significantly increased compared to WT mice, we do not consider this a very likely interpretation. In other words, it seems that efficient control of the local infection is critical to the anti-inflammatory effect of the recruited CD8 T cells.

We further demonstrated that, following lethal infection, CXCR3-dependent recruitment of effector ZIKV-specific CD8 T cells to the CNS is important for efficient control. In a recent report, it has been shown that, at the peak of lethal encephalitis, ZIKV infects all major cells of the CNS and in particular oligodendrocytes in the cortex, hippocampus, and thalamus of WT mice (62). Failure of effector CD8 T cells to express CXCR3 often results in their inefficient recruitment to the right compartment of the inflamed tissue. This was first observed for lymphocytic choriomeningitis virus (LCMV) infected animals (63); however, a similar observation has been made for WNV. CXCR3-deficient mice have been found unable of controlling WNV infection of the CNS due to the inability of virus-specific CD8 T cells to be correctly positioned within the cerebellar department, which were instead found to accumulate in other regions of the CNS (64). In our study, we observed that the total numbers of CD8 T cells as well as ZIKV-specific CD8 T cells were significantly reduced in the CNS of CXCR3-deficient mice on day 2 post i.c. infection. We do not know precisely where these cells are located, but the protection was inferior compared to that in WT mice. In general, CXCR3 regulates trafficking of activated cells via interaction with CXCL9, CXCL10 and/or CXCL11 in the inflamed CNS (65–67). People acutely infected with ZIKV and tick-borne encephalitis (TBE) virus, display elevated levels of CXCL10 in their serum (68, 69). In addition, in mouse models of DENV and WNV, CXCL10-deficiency was associated with a more severe outcome of disease following i.c. challenge (64, 70). For DENV this deficiency was not associated with decreased levels of cellular recruitment to the CNS, but rather with reduced antiviral activity in the absence of CXCL10 (71). In our study, CXCL10-deficient mice displayed similar levels of protection to WT mice, suggesting that for ZIKV, CXCR3-dependent recruitment is perhaps guided primarily by other ligands such as CXCL9 (CXCL11 represents a pseudogene in B6 mice).

## Conclusion

Our data convincingly show that CXCR3-dependent recruitment of ZIKV-specific CD8 T cells to the site of CNS infection early after ZIKV invasion leads to a marked reduction of viral replication inside the infected CNS. Therefore, in relation to preventing severe human disease, which most likely does not involve early CNS invasion, our results highlight the value of including priming of CD8 T cells in vaccination approaches against ZIKV virus.

## Data Availability Statement

The datasets generated for this study are available on request to the corresponding author.

## Ethics Statement

The animal study was reviewed and approved by the Danish Animal Experiments Inspectorate, Ministry of Justice, permission number 2015-15-0201-00623.

## Author Contributions

LN, SB, AS, JC, and AT designed the study and interpreted the data. LN, ASS, and MB performed the experiments and analyzed the data. LN and AT drafted the manuscript. All authors contributed to the article and approved the submitted version.

## Conflict of Interest

The authors declare that the research was conducted in the absence of any commercial or financial relationships that could be construed as a potential conflict of interest.
