# Supplementary material for: Effector CD8 T Cell-Dependent Zika Virus Control in the CNS: A Matter of Time and Numbers
Source: Front Immunol. 2020 Aug 18;11:1977. doi: 10.3389/fimmu.2020.01977 (PMC7461798; doi:10.3389/fimmu.2020.01977)
Supplement: Supplementary file 1 [file Data_Sheet_1.PDF]

Supplementary Material

Supplementary Figures

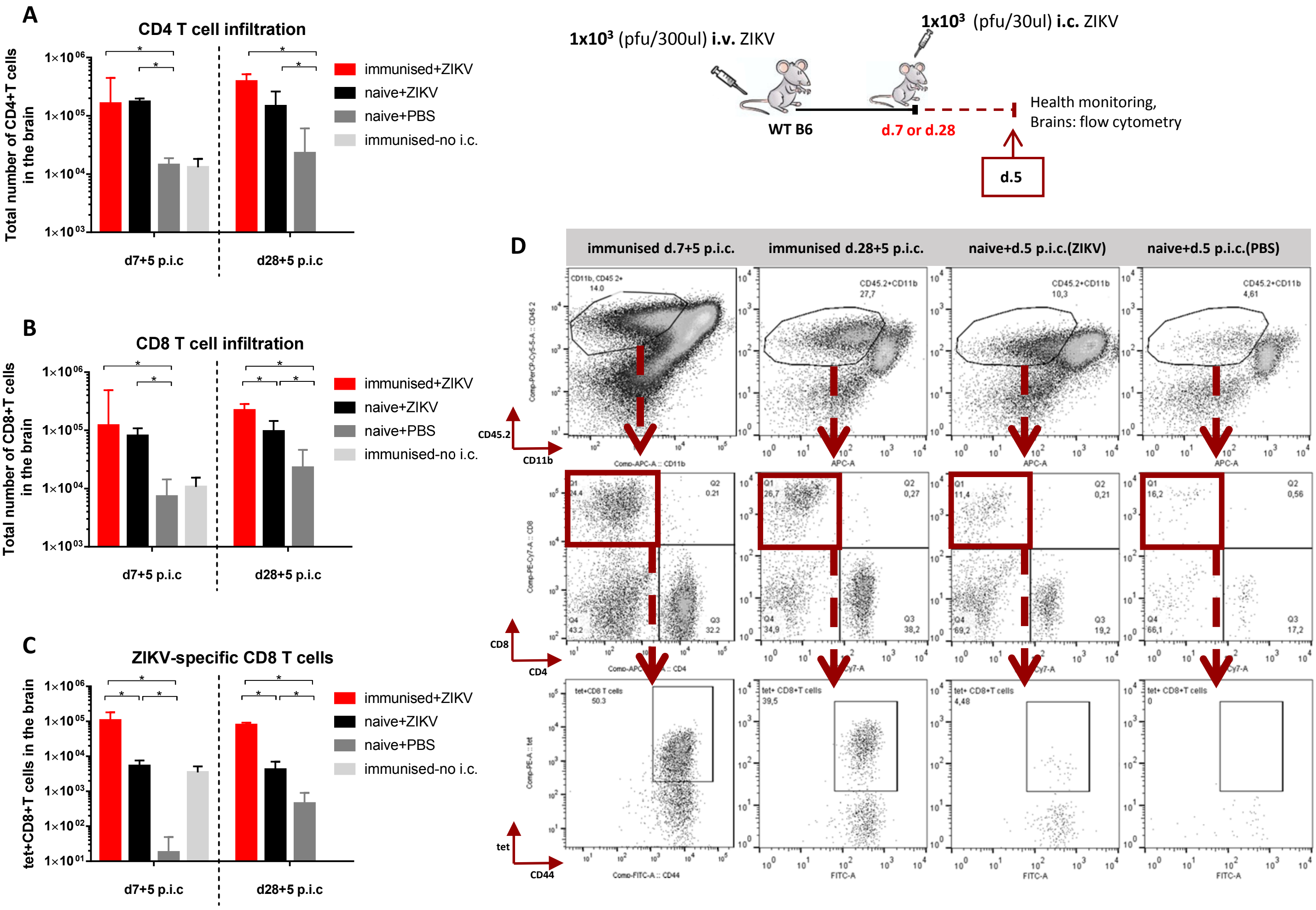

Supplementary Figure 1. Similar patterns of cellular infiltration in the brain week1+5 post i.c. vs week4+5 post i.c. WT C57BL/6 mice were inoculated with 1x10^3 pfu ZIKV i.v. and either 1 or 4 weeks later, these mice along with naive controls, were challenged with 1x10^3 pfu ZIKV i.c. A group of naive mice injected i.c. with PBS and a group of immunized mice not challenged were also included for control. Health status was monitored daily and on day 5 post i.c. challenge, brains were removed and the total number of CD4 T cells **(A)**, CD8 T cells **(B)**, and ZIKV-specific CD8 T cells **(C)** were determined via flow cytometry. Representative flow plots are included **(D)**. The results represent the group medians +/- ranges. n=4-5/group. *p< 0.05
